# Indonesia's new health law: lessons for democratic health governance and legislation

**DOI:** 10.1016/j.lansea.2024.100390

**Published:** 2024-03-16

**Authors:** Nico Gamalliel, Ahmad Fuady

**Affiliations:** a Evidence-Based Health Policy Center, Indonesian Medical Education and Research Institute, Faculty of Medicine, Universitas Indonesia, Jakarta, Indonesia; b Department of Community Medicine, Faculty of Medicine, Universitas Indonesia, Jakarta, Indonesia

A new health law has been introduced in Indonesia, a country with a population of 278-million and multiple burdens of health—backlog of infectious diseases, malnutrition, and maternal mortality, rapidly increasing non-communicable diseases, and globalization-related emerging diseases. This law, known as law No. 17 of 2023 on Health,^1^ was enacted following the Indonesian Health Transformation Plan, encompassing six key pillars: the transformation of primary health care, referral health care, health resilience, health financing, human resources, and health technology. As it is crucial to acknowledge the Indonesian government's commitment to transforming the health system and solving pressing issues in the country's healthcare system, including disparities in healthcare access, workforce, and capacity, the health law has sparked contentious public debates, which could offer valuable insights into current health policy.

This law adopts an ‘omnibus’ approach (legislative method that could change and repeal various existing regulations^2^), merging and amending 13 existing health-related laws into a single 458-article law, in less than twelve months. The same approach was previously applied to the Indonesian Job Creation Law and triggered country-wide protests. This ‘omnibus’ method is mostly practiced in countries with common law/Anglo-Saxon system,^3^ and is often perceived as problematic and non-inclusive.

The health legislation could serve as modality to formalize commitments and goals, establish the fundamental aspects of health system architecture, and facilitate multisector cooperation to achieve the health system goals.^4^ However, the only-two-week public hearings held by the Indonesian government also met with skepticism as formality process of “ticking a checkbox” rather than a substantial review process. This contributed to increased resistance and distrust among the public and healthcare professionals.^5^ The process did not show a constructive relationship and robust public involvement, which is vital to strengthen democratic processes and reinforce accountability, transparency, equity, and therefore the ability to tailor solutions to meet community needs. Instead of doing so, it is important in every legislation process to find the common ground between interests to foster greater and meaningful public participation, aiming at achieving shared ownership of both the process and the outcomes.

The law also removed the mandate to allocate 5% of the national budget and 10% of the subnational budget for health programs, leaving the political commitment for health budget not guaranteed.^6^^,^^7^ The government argued this mandate was not efficient, as the funding allocation had not matched actual needs. Additionally, of the approximately USD594 million transferred annually from the central to the subnational governments, only 61% of these funds had been properly utilized. To enhance efficiency, the plan is to allocate the health funding exclusively to approved and well-planned programs through Health Master Plan (*Rencana Induk Bidang Kesehatan,* RIBK), which has not been developed.^1^

While political power and health administration are decentralized to subnational authorities across 38 provinces and 514 cities/districts, the decentralization of the health system, coupled with a significant gap in health system capacity,^8^ may exacerbate inequalities. These challenges would become even more pronounced after the massive governors' and regents'/mayors' elections, potentially resulting in health issues being neglected or reduced to mere political commodities during political campaigns. To address these challenges effectively, it is crucial to develop a comprehensive Health Master Plan based on an evidence-based approach. This plan should involve proper, robust, clear, and direct instructions, as well as meaningful participation in its creation.

In addition, the law seemed to overlook other vital issues, such as enforcing tobacco control,^7^ which presented a paradox of how the government did not exhibit the expected level of commitment when addressing essentials health issues. The lack of planning and dialogue, and conflicts following them have made the new health law become another scar in the country's democracy and health system history.

The dynamics surroundings this law should serve as valuable learning experience for Indonesians and the world. From a global perspective, as tackling global health challenges eventually should involve each country's health system, the health legislation saga of “the biggest invisible country” provide more understanding of how health system could interplay with a country's regulatory and governance system. The commitment to build and develop health systems would be best to be in tune with the commitment to value evidence-based approach, good governance, and meaningful public participation.

## Contributors

NG did the conceptualization, writing, and finalization of the article. AF did the conceptualization, writing, and review of the article.

## Declaration of interests

We declare no competing interests.
